# A systematic review and meta-analysis on adoption of WHO-recommended infant feeding practices among HIV positive mothers in Ethiopia

**DOI:** 10.1186/s12884-021-03662-3

**Published:** 2021-03-08

**Authors:** Amare Belachew Dagnew, Mulat Dagnew Teferi

**Affiliations:** 1 College of Medicine and Health Sciences, Bahir Dar University, Bahir Dar, Ethiopia; 2 College of Medicine and Health Sciences, University of Gondar, Gondar, Ethiopia

**Keywords:** Infant feeding options, Exclusive breastfeeding, HIV positive mothers, Prevalence, Ethiopia

## Abstract

**Background:**

The prevalence of the World Health Organization (WHO) recommended infant feeding practices for HIV exposed infants is low in developing countries. There is no nationwide representative study was done in Ethiopia. Therefore, this study aimed to assess the pooled prevalence of WHO-recommended infant feeding practices among HIV-positive mothers in Ethiopia.

**Methods:**

EMBASE, PubMed, Google Scholar, CINHAL, Web of Science, Cochrane library, and hand searches of references were extensively searched to find out the primary articles. This study was included in all primary articles published in peer review journals regarding the recommended infant feeding practices in Ethiopia. Reviewers were used a standardized Microsoft Excel format to extract the data and analyzed it with Stata 11 version software. The pooled prevalence of recommended infant feeding practices among HIV exposed infants was estimated by a random-effect model. The sources of variation between the studies were identified by the *I* ^*2*^ statistics test. Furthermore, the source of heterogeneity was checked by subgroup and meta-regression analyses. Sensitivity analysis was also carried out for included articles to identify extreme values that affect the outcome of pooled results.

**Results:**

A total of twenty-one articles were included in this study. The random effect pooled prevalence of WHO-recommended infant feeding practices in Ethiopia was 82.76% (95% Confidence Interval [CI]: 75.4, 90.11) with the heterogeneity of I^2^ = 93.7 with a value of *p* < 0.001. The subgroup analysis result showed that the highest prevalence of WHO-recommended infant feeding practices was observed in the retrospective cohort study design, 89.45%, and the lowest prevalence was found in cross-sectional studies, 80.67%. Mothers who disclosed their HIV serostatus to their spouses OR = 2.88(2.27, 3.66) and attended antenatal care visits OR = 4.62(3.13, 6.83) were more likely to follow the WHO-recommended infant feeding practices than their counterparts.

**Conclusion:**

Two out of ten HIV exposed infants received mixed feeding in Ethiopia. Health professionals should support and counsel HIV positive mothers to disclose their HIV serostatus to their spouses and advertisements in general or community health workers can get this message out to encourage using antenatal care services during the pregnancy period were recommended to increase the adoption of WHO recommended infant feeding practices and decrease their infant’s risk of morbidity, including HIV infection.

## Background

More than 37.9 million people are living with HIV in the globe and 1.7 million of them are children. A majority of these children are living in sub-Saharan Africa [1] and over one-hundred thousand children are living in Ethiopia [2]. The majority (90%) of the children have acquired HIV infection through vertical transmission and breastfeeding is the main route of transmission when there was no PMTCT care given to the exposed ones [1]. The magnitude of vertical HIV infection in Ethiopia due to breastfeeding to 18–24 months was 28% [2].

WHO has forwarded recommendations for the HIV-exposed infants to feed either exclusive breastfeeding or replacement feeding for the first 6 months of life with highly maternal antiretroviral therapy (ART) and ART prophylaxis for exposed neonates, followed by complementary feeding with continued breastfeeding through 12–24 months of age [3]. Although there is a risk of HIV infection through breastfeeding, the risk of mortality or morbidity also high if infants are taking replacement feedings [3]. In many resource-limited settings, infants who were not breastfed were up to six times more likely to die from diarrheal illnesses, malnutrition, and pneumonia [3]. Thus, breastfeeding is important for protection against other child infections, mal-occlusion helps to increase intelligence, prevent early childhood obesity and diabetes [4].

Different studies have assessed the prevalence of WHO-recommended infant feeding practices and associated factors among HIV exposed infants in Ethiopia [5–22]. The highest adoption of WHO-recommended infant feeding practices was found from research done in Addis Ababa, 98.5% [10] while the lowest level of practice was from Wolega, 36.4% [21]. These findings showed that there is great variability in the prevalence of WHO-recommended infant feeding practices across the country. Antenatal care visits [5–18], disclosure of HIV status to her spouse [5–7, 9, 11, 14, 15, 18], husband support [5–7], occupational status [5–7], place of delivery [4–6], insufficient milk [4], knowledge of mothers on infant feeding options [7, 10, 11], educational status of mothers [5–7, 9], being on ART [6], were some of the factors associated with the adoption of WHO-recommended infant feeding practices. Among these factors, antenatal care visits and disclosure of their HIV status to their spouses have selected due to the variability of their odds ratio in different study areas. Antenatal care visits odds ratio was high in a study done in Northwest Ethiopia OR = 11.2(5.34, 25.26), while lower odds ratio was seen in Debre Markos, OR = 2.12(1.12, 4.01) [6, 14]. Besides, the association of disclosure of mothers HIV status to their spouses and recommended infant feeding practices were supported with studies were done in [5, 6, 9, 14, 15], while studies were done in Addis Ababa and the southern part of Ethiopia did not support this finding [10, 18].

Although many different studies were done regarding the adoption of WHO-recommended infant feeding practices, there are fragmented and no representative study was done which determined the pooled prevalence of WHO-recommended infant feeding practices and its association with antenatal care visits and disclosure of mothers’ HIV status to their spouses in Ethiopia. Thus, this study was aimed to estimate the pooled prevalence of WHO-recommended infant feeding practices and its association with antenatal visits and disclosure of status to their spouse in the context of Ethiopia. This study finding would be an input for program planners and policymakers working on the area of PMTCT.

## Method

### Databases and searching strategy

The preferred reporting item for systematic reviews (PRISMA) guidelines was followed for this review [23]. All studies that reported recommended infant feeding practices were searched through CINAHL, PubMed, Google Scholar, EMBASE, and Cochrane library. The searching terms were “Exclusive breastfeeding”, “Exclusive replacement feeding”, “prevalence “Incidence”, “Burden”, Factors”, “Predictors”; “Recommended infant feeding practices”, “Determinants”, “HIV exposed infants”, Formula feeding and “Ethiopia”. The key words and the boolean operators term such as “OR” or “AND” or AND/NOT or AND, NOT were used in combination and separate manner. The initial search was done on October 19, 2019. The search was re-done on February 16, 2021, and no further papers were found.

### Inclusion and exclusion criteria

Studies have been done on the prevalence of WHO recommended infant feeding practices and its associations with disclosure of mother’s HIV status to spouse and antenatal visits in Ethiopia, with cross-sectional, cohort study designs, and having full texts were included in this review. But, studies reported on single case study designs, published in books, and qualitative studies were excluded.

### Operational definitions

WHO recommended infant feeding practices: HIV-positive mothers must practices either exclusive breastfeeding with maternal ART and appropriate ART prophylaxis for children or exclusive replacement feeding until the child reaches to the age of six-months old [5].

### Data extractions and quality assessment

Joanna Briggs Institute Meta-Analysis of Statistics Assessment and Review Instrument (JBI-MAStARI) was used to critically appraise the quality of paper [24]. Clear definition criteria were included for the inclusion of the selection of the study participants, sample, outcome interest, confounding factors, statistical analysis methods, and consistent measurement of outcome variables [24]. The criteria were dealing before the extraction of the data. The quality of included studies was taken a mean score value of two independent reviewers. Components used to extract the data were primary author, year of the publication, study area, sample size, study design, region of the study, prevalence of recommended infant feeding practices, age of child, and response rate. Article inclusion, data extraction, and compared results were independently carried out by the reviewers. Finally, the disagreement was resolved by a consensus of the reviewer.

### Data analysis

Data were extracted using Microsoft Excel and exported to Stata version 11 software for analysis. The variation between studies was assessed by the I^2^ statistic test [25]. Laird’s random-effects model was used to estimate the pooled prevalence of WHO-recommended infant feeding practices among HIV exposed infants in Ethiopia [26]. Subgroup analysis was done by study design to minimize the random variations between the point estimates of the primary studies. Furthermore, univariate meta-regression analysis was undertaken using sample size, study setting, study design, region, and publication date. The Logs odd’s ratio was used to determine the association between WHO recommended infant feeding practices with antenatal visits and disclosure of HIV status to her spouses.

### Publication bias

Fill trim analysis and Egger tests were done for assessing publication bias [27].

## Results

**Fig. 1:**
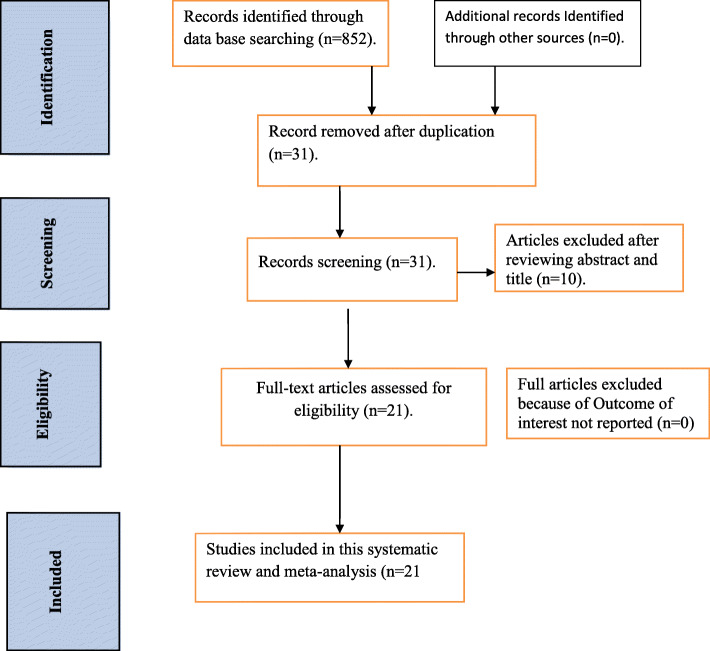
Flowchart diagram to reflect the selection of studies for systematic review and Meta analysis of the prevalence of WHO-recommended infant feeding practice and its association with antenatal visits and disclosure of their status in Ethiopia

A total of 852 research articles with regard to WHO recommended infant feeding practices in Ethiopia were retrieved through SCOPUS, PubMed, Google Scholar, Web of Science, Cochrane library, and African journal of health sciences. Of this, 821 were excluded due to duplication and irrelevant and 10 articles were excluded after reviewing the abstract and title of the study. Finally, 21 studies were included in this review (Fig. 1).

### Characteristics of the original studies

As described in Table 1, a total of 6575 HIV positive mothers enrolled in the prevention of mother to child transmission (PMCT) were included to estimate the pooled prevalence of WHO recommended infant feeding practices. Almost all (99.31%) mothers practiced WHO-recommended infant feeding practices from a study done in Oromiya and Amhara regions [29, 30] whereas the lowest prevalence (34.6%) was reported from a study done in the Oromia region [21]. In this meta-analysis, five out of nine regions of the country and 21 studies were included. From included studies, seven studies were from the Amhara region [5, 6, 8, 14, 22, 28, 30], six studies were from the Oromia region [6, 9, 13, 19, 21, 29], four studies were from Addis Ababa city [10–12, 17], three studies were from the SNNP region [15, 18, 20], and the remaining one study was from Tigray region [16]. A total of sixteen studies with cross-sectional and five studies with cohort study designs were included in this review (Table 1).

**Table 1:** Descriptive summary of 21 studies included in the meta-analysis of the prevalence of recommended infant feeding practices among HIV positive mothers in Ethiopia, 2019

Authors /Year of publication	Region	Study setting	Study design	Response rate	Sample size	EBF/ERF	Prevalence	Quality
Muluye D et al., 2012 [5]	Amhara	Institutional based	cross sectional	100%	209	187	89.5	Low score
Wakwoya EB et al., 2016 [6]	Amhara	Institutional based	cross sectional	100%	260	223	85.8	Low score
Mengistu YG et al., 2016 [7]	Oromia	Institutional based	cross sectional	100%	327	277	84.7	Low score
Sendo EG et al., 2018 [8]	Amhara	Institutional based	cross sectional	100%	230	205	89.13	Low score
Ejara D et al., 2018 [9]	Oromia	Institutional based	cross sectional	97%	283	260	91.7	Low score
Maru Y et al., 2009 [10]	Addis Ababa	Institutional based	Cross sectional		209	206	98.5	Low score
Mukerem M et al., 2012 [11]	Addis Ababa	Institutional based	Cross sectional	100%	384	271	73	Low score
Mirkuzie AH et al., 2018 [12]	Addis Ababa	Institutional based	Retrospective	100%	769	691	89.9	Low score
Obsa S et al., 2018 [13]	Oromia	Institutional based	Retrospective	100%	492	396	80.5	Low score
Esubalew F et al. 2018 [14]	Amhara	Institutional based	Cross sectional	100%	420	265	63.1	Low score
Modjo KE et al. 2015 [15]	SNNP	Institutional based	Cross sectional	99.3%	436	285	65.4	Low score
Girma Y et al. 2014 [16]	Tigray	Institutional based	Cross sectional	100%	207	194	93.7	Low score
Negash TG et al. 2016 [17]	Addis Ababa	Institutional based	Cross sectional	100%	384	319	83	Low score
Mengiste A et al. 2016 [18]	SNNP	Institutional based	Cross sectional	100%	87	56	64.4	Low score
Wudineh F et al. 2016 [19]	Oromia	Institutional based	Cross sectional	100%	382	347	90.84	Low score
Tadele T et al. 2016 [20]	SNNP	Institutional based	Retrospective	100%	457	390	85.33	Low score
Bayissa ZB et al. 2016 [21]	Oromia	Institutional based	Cross sectional	100%	392	136	34.69	Low score
Brhan Z et al. 2014 [22]	Amhara	Institutional based	Retrospective	100%	434	402	92.6	Low score
Dagnew AB et al., [28]	Amhara	Institutional based	Cross sectional	100%	213	178	85.56	Low score
Demissie DB et al., 2016 [29]	Oromia	Institutional based	Cross sectional	100%	145	146	99.31	Low score
Moges NA. et al., 2017 [30]	Amhara	Institutional based	Cohort	100%	305	303	99.34	Low score

*NB* Outcome (recommended infant feeding practices), *IFP* Infant feeding practices

### Publication bias

**Fig. 2:**
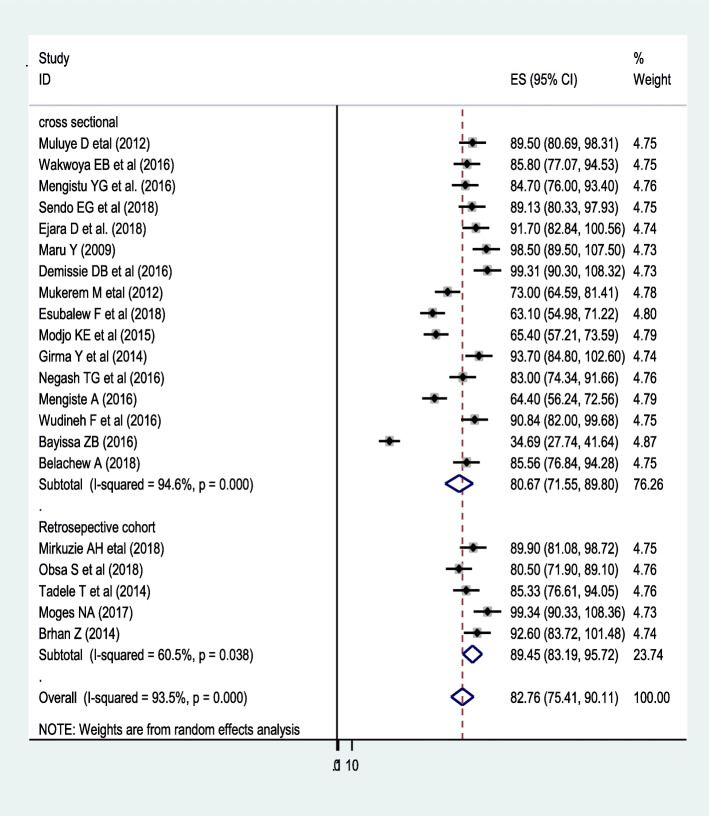
Forest plot of prevalence of recommended infant feeding options among HIV positive mothers in Ethiopia, 2019 (*n* = 21)

The publication bias was assessed by the Egger test. The result was − 0.20 (95% CI = 0.33, −.07) *p* = 0.06 which indicates there was not a statistically significant publication bias (Fig. 2).

### Overall pooled prevalence of the adoption of WHO recommended infant feeding practices

**Fig. 3:**
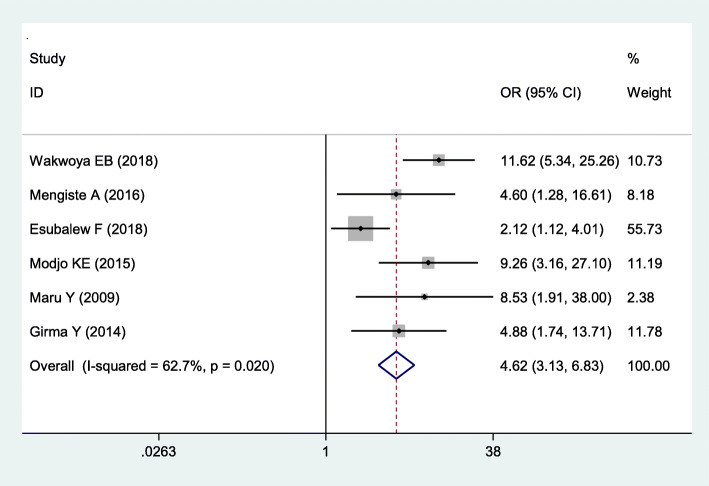
The pooled odds ratio of the association between antenatal care visit and WHO-recommended infant feeding practice in Ethiopia

A total of twenty-one studies were included in this review and the overall pooled prevalence of WHO-recommended infant feeding practices in Ethiopia was 82.76% (CI: 75.4, 90.11). A severe heterogeneity was observed across the studies (I^2^_=_93.5, *p*-value = < 0.001). Subgroup analysis was done by region, sample size, and publication year and none of them showed statically significance except study design. Accordingly, a slightly higher level of WHO recommended infant feeding practices was observed in cohort studies (89.45%) while lower in cross-sectional studies (80.67%) (Fig. 3).

### Meta-regressions

The univariate meta-regression analysis was done with study design, publication date, sample size, region, and study design to identify the source of heterogeneity between the studies. But, none of them were statistically significant (Table 2).

**Table 2:** Univariate Meta-regression analysis of studies on WHO recommended infant feeding practices in Ethiopia, 2019

		Co-efficient	*P*-value	95% confidence interval
Year of study		0.023	0.99	− 17.71, 17.76
Sample size		0.0004	0.99	− 0.27, 0.276
Region	Addis Ababa	−0.01	0.99	−3.17, 3.15
	Amhara	−0.004	0.99	−3.36, 3.35
	Oromia	0.09	0.95	−3.30, 3.50
	Tigray	−0.02	0.994	−4.79, 4.76
	SNNP	1		
Study design	Cross-sectional	1		
	Cohort	−0.025	0.991	−1.908, 1.85

### Association between antenatal care visits and safe infant feeding practice

**Fig. 4:**
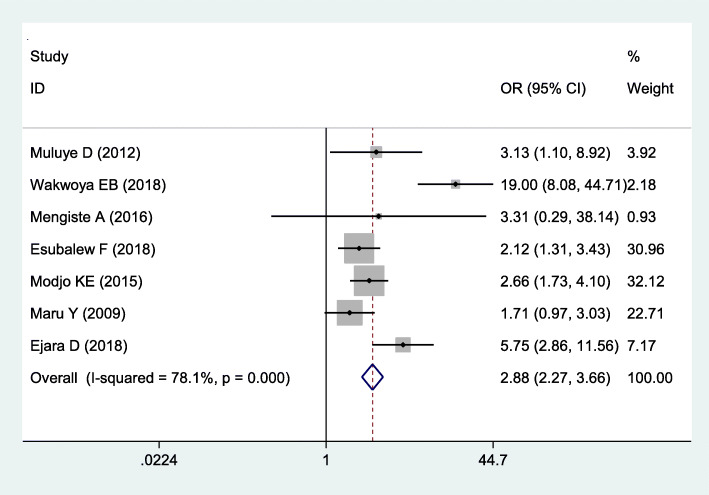
The pooled odds ratio of the association between disclosure of their status to the spouse with WHO-recommended infant feeding practices in Ethiopia

Six studies were included to examine the association between antenatal care visits and WHO recommended infant feeding practices [6, 11, 14–16, 18]. The result showed that there is a significant association between antenatal care visits and WHO-recommended infant feeding practices. HIV positive pregnant mothers who attended antenatal care visits were 4.62 times more likely to adopt or follow WHO-recommended infant feeding practices (OR: 4.62, 95% CI: 3.13, 6.83) than counterparts. Moderate heterogeneity (I = 62.7% and *p*-value< 0.02) was observed across the included studies. The result of the Egger test indicated that there was a statistically significant publication bias (*p < 0.001*) (Fig. 4).

### Association between disclosure of HIV status and WHO recommended infant feeding practices

Seven studies were included to determine the association between disclosure of mother’s HIV serostatus to their spouses and WHO-recommended infant feeding practices [5, 6, 9, 11, 14, 15, 18]. The result indicated that the pooled odds ratio of disclosure of HIV status was significantly associated with the WHO-recommended infant feeding practices (OR = 2.88 (2.27, 3.66). Due to the presence of severe heterogeneity across the studies (I^2^ = 78.1% and *p*-value =0.000), a random-effects model was employed to estimate the pooled effect. Moreover, Egger’s test was done to assess publication bias and it showed that not statistically significant, *P* = 0.149.

## Discussion

Though the World Health Organization has recommended HIV positive mothers to practice either exclusive breastfeeding or replacement feeding for their exposed infant for the first 6 months of life, the practice was not satisfactory. The overall pooled prevalence practice of HIV positive mothers towards the adoption of WHO-recommended infant feeding practices in this study was 82.76% (CI: 75.4, 90.11). This finding is similar with studies done in Uganda, 79.4% [31], Nigeria, 87% [32], and Kenya, 88.1% [33]. However, it is higher than studies done in India, 67.1% [34], and Singapore, 62.02% [35]. The possible explanation might be due to difference in socio-demographic characteristics, study design, and cultural variability between the studies.

The result of this finding is lower than studies done in South Africa, 90% [36], Lesotho 97% [37], and South Sudan, 96% [38]. This difference might be due to culture variation related to feeding practices, socio-economic variation, knowledge gaps regarding exclusive breastfeeding, quality or time spent with mothers; duration or frequency of counseling by health care workers, and provider training or interest in providing infant-feeding option counseling services.

In sub-group analysis, the pooled prevalence of recommended infant feeding practice varies with study designs. Studies done with cohort study design show a slightly higher prevalence of recommended infant feeding practices, 89.45% compared to those conducted with cross-sectional study designs, 80.67%. This difference could be the cohort study design may better estimate the prevalence of recommended infant feeding practices.

The WHO recommended infant feeding practices were influenced by different factors. Mothers who attended antenatal care visits (> = 4) were 4.62 times more likely to practice recommended infant feeding practices than their counterparts. This finding is in line with studies done in Rwanda [39], Odisha [40], Botswana [41], and other sub-Saharan Africa countries [42]. This may be due to mothers receiving information and knowledge regarding infant feeding options during their ANC visits. Besides, the close interaction of mothers with health workers may increase psychosocial support regarding infant feeding practices.

Disclosure of the mother’s HIV serostatus to their spouse is also another determining factor of WHO-recommended infant feeding practices. Mothers who disclosed their HIV serostatus to their spouses were 2.28 times more likely to practice WHO recommended infant feeding practices than those who did not disclose. This finding is consistent with studies done in Nigeria [43] and South Africa [44, 45]. This might be due to mothers who disclosed their HIV serostatus to their spouses are more likely to receive comfort, support from their husbands, and feel relieved [46]. Additionally, it might have psychological benefits as they do not hide while formula feeding; disclosure may enable them to have a favorable attitude towards exclusive breastfeeding practice. Hence, mothers can practice freely when feeding their infant without fear and helps to reduce the practice of mixed feeding.

### Strengths and limitations

Since it is the first review study, it may be used as a baseline to do further experimental research studies. The magnitude of outcome may be affected by other confounding variables because of recall bias and age-group of the children assessed.

## Conclusions

Approximately two out of ten HIV positive mothers did not adopt WHO recommended infant feeding practice in Ethiopia. Health professionals should support and counsel HIV positive mothers to disclose their HIV serostatus to their spouses and encourage them to visit antenatal care services. Therefore, adopt WHO recommended infant feeding practices help to save their infants from the risk of morbidity as well as for the free survival of viral infections.

## Data Availability

Data will be available upon request of the corresponding author.

## Abbreviations

ANC: Antenatal Care
EDHS: Ethiopian Demographic and Health Survey
HIV: Human Immune Deficiency Virus
MTCT: Mother to Child Transmission
OR: Odds Ratio
PMTCT: Prevention of Mother to Child Transmission
WHO: World Health Organization
